# Potential Use of Biological Proteins for Liver Failure Therapy

**DOI:** 10.3390/pharmaceutics7030255

**Published:** 2015-08-31

**Authors:** Kazuaki Taguchi, Keishi Yamasaki, Hakaru Seo, Masaki Otagiri

**Affiliations:** 1 Faculty of Pharmaceutical Sciences, Sojo University, 4-22-1 Ikeda, Nishi-ku, Kumamoto 862-0082, Japan; E-Mails: k-taguchi@ph.sojo-u.ac.jp (K.T.); kcyama@ph.sojo-u.ac.jp (K.Y.); seo@ph.sojo-u.ac.jp (H.S.); 2 DDS Research Institute, Sojo University, 4-22-1 Ikeda, Nishi-ku, Kumamoto 862-0082, Japan

**Keywords:** albumin, lactoferrin, erythropoietin, α_1_-acid glycoprotein, gelatin, hemoglobin, nanomedicine, drug delivery

## Abstract

Biological proteins have unlimited potential for use as pharmaceutical products due to their various biological activities, which include non-toxicity, biocompatibility, and biodegradability. Recent scientific advances allow for the development of novel innovative protein-based products that draw on the quality of their innate biological activities. Some of them hold promising potential for novel therapeutic agents/devices for addressing hepatic diseases such as hepatitis, fibrosis, and hepatocarcinomas. This review attempts to provide an overview of the development of protein-based products that take advantage of their biological activity for medication, and discusses possibilities for the therapeutic potential of protein-based products produced through different approaches to specifically target the liver (or hepatic cells: hepatocytes, hepatic stellate cells, liver sinusoidal endothelial cells, and Kupffer cells) in the treatment of hepatic diseases.

## 1. Introduction

Scientific advances such as protein engineering, biochemical analysis, and analytical instrumentation techniques have revealed new information regarding biological proteins, including detailed information regarding the structure and biological functions of these proteins. Taking advantage of these data, certain biological proteins, such as albumin, fibrinogen, and immunoglobulin, are now routinely used in clinical situations as the gold standard for the treatment of severely ill patients. Furthermore, they have unlimited potential for further use in a variety of clinical situations. A number of studies have reported on the innate biological characteristics of biological proteins, and protein-based products are currently under development. One of the benefits of using proteins for medications is that they have higher biocompatibility and biodegradability than polymeric drugs that are based on synthetic polymers. Thus, biological proteins can be useful as biologics and/or carriers for other bioactive agents. In fact, an albumin–paclitaxel nanoparticle (Abraxane^®^), which has been approved for the treatment of breast, lung, pancreatic, and small cell lung cancers worldwide, was designed and developed using albumin to both increase the water solubility of paclitaxel (innate biological characteristic of albumin) and control the size of particles to permit them to accumulate in the solid tumor (carrier) [1,2]. Many protein-based drugs are currently on the market or under clinical/preclinical development.

The liver is well known for its ability to take up high amounts of exogenous substances, because liver Kupffer cells make up approximately 80% of the macrophages in the entire human body [3]. Such cells take up large foreign molecules, including therapeutic compounds. However, the activity of Kupffer cells becomes altered under the conditions of liver disease, resulting in a decrease in the hepatic distribution of exogenous substances, depending on the type and severity of the hepatic disease [4]. Furthermore, the four major cell types (hepatocytes, hepatic stellate cells (HSCs), liver sinusoidal endothelial cells, and Kupffer cells) are all present in the liver, and target cell types for therapeutic intervention varies with the hepatic diseases [5,6,7]. In the past half century, many protein-based products for the treatment of acute and chronic liver failure were designed and developed by using the innate characteristics of each protein (Table 1). The present review attempts to introduce examples of prospective protein-based products produced through different approaches to specifically target the liver (or hepatic cells) for use in the therapeutic treatment of acute and chronic liver failure.

## 2. Albumin-Based Products

**Figure 1:**
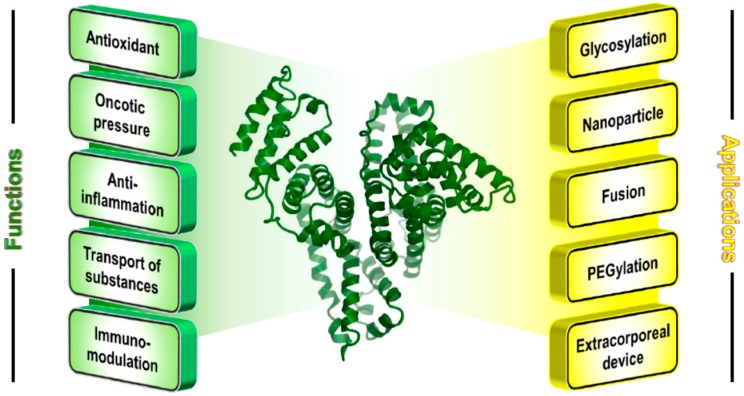
Crystal structure of human serum albumin (**center**), and its function (**left**) and modifications for medical/pharmaceutical applications (**right**). The crystal structure was prepared using the CueMol software and the structural coordinates of PDB 1BM0.

Albumin is an approximately 67 kDa monomeric protein produced by hepatocytes and has been shown to be nontoxic, non-immunogenetic, biocompatible, and biodegradable, thus making it a versatile protein with potential for use in the production of protein-based products (Figure 1). In addition, albumin has many innate functional properties, such as the binding and transport of many endogenous and exogenous substances, antioxidant functions, immuno-modulation, and anti-inflammatory activity (Figure 1) [8,9]. These functions of albumin, human serum albumin (HSA), or bovine serum albumin (BSA) make it the most widely-used protein for the chemical or genetic development of albumin-based products, as discussed below.

**Table 1:** Brief summary of biological and modified proteins for liver failure therapy that are in different stages of development.

Product	Features	Product	Features
**Albumin**		**Erythropoietin (EPO)**	
Recombination	Marketed approval	Unmodification	Animal study
Mannosylation	Animal study	EPO with G-CSF	Clinical study
M6P-modification	Animal study		
Nanoparticle	Animal study	α_1_-**Acid glycoprotein (AGP)**	
Fusion with Trx	Animal study	Unmodification	Animal study
Fusion with IFN-α	Clinical study		
PEG modification	Animal study	**Gelatin**	
MARS^®^	Clinical study	Nanoparticle	Animal study
**Lactferrin (Lf)**		**Hemoglobin (Hb)**	
Unmodification	*In vitro* study	CO-bound liposomal Hb	Animal study
	Animal study		
PEG modification	Animal study	Hb-ribavirin	Animal study

M6P, mannose-6-phosphate; Trx, thioredoxin-1; IFN-α, Interferon-α; PEG, polyethylene glycol; MARS^®^, molecular adsorbents recirculatory system; G-CSF, granulocyte colony stimulating factor; CO, carbon monoxide.

### 2.1. Mannosylation

The use of mannosylated albumin is one of the suitable strategies for liver-selective targeting via a mannose receptor. In a previous study, we genetically prepared mannosylated-recombinant HSA (Man-rHSA) using a *Pichia pastoris* expression system [10]. This highly Man-rHSA was shown to be selectively distributed in the liver, which is mediated by mannose receptors on Kupffer cells. Taking advantage of this liver-specific distribution characteristic, we succeeded in using this highly Man-rHSA as a nitric oxide (NO) carrier in the treatment of hepatic ischemia/reperfusion injury [10]. Interestingly, Man-rHSA was selectively targeted to mannose receptors (CD206^+^) on CD68^+^ Kupffer cells [11]. This means that Man-rHSA has great potential for use as a drug carrier for hepatopathy related to reactive oxygen species (ROS) because CD68^+^ Kupffer cells can produce relatively high levels of ROS, which play a major role in the progression of liver pathologic conditions [12]. In fact, polythiolated- and mannosylated-rHSA (SH-Man-rHSA) showed excellent hepatoprotective action against both concanavalin A-induced and acetaminophen-induced hepatitis [11].

During the course of a liver disease, HSCs transdifferentiate into extracellular matrix-producing, highly proliferative myofibroblasts that promote hepatic fibrogenesis [13]. Thus, HSCs are considered to be key target cells for fibrosis therapy. The mannose-6-phosphate (M6P) receptor is upregulated in HSCs, resulting in a high density of these receptors on the cell surface [14]. For drug targeting to HSCs, many researchers coupled antifibrotic drug to M6P-modified HSA, resulting in the suppression of hepatic fibrosis [15,16,17,18]. These findings show that M6P-modified HSA holds promise as a selective targeting carrier for HSCs.

### 2.2. Nanoparticles

Albumin nanoparticles are ideal for encapsulating lipophilic drugs within them due to the high protein binding of lipophilic drugs to albumin, and a large variety of protocols for preparing albumin nanoparticles have been established [19,20]. There are many reports that show the potential for such materials for use as carriers for liver-targeting drug delivery. Li *et al.* prepared sodium ferulate (SF)-loaded BSA nanoparticles using a desolvation procedure and showed a much higher hepatic delivery of SF in SF-loaded BSA nanoparticles than in SF solutions [21]. Furthermore, they prepared SF-loaded M6P-modified BSA nanoparticles (SF-M6P-BSA nanoparticles) for targeted drug delivery to HSCs [22]. As the result, the SF-M6P-BSA nanoparticles were taken up specifically by HSCs and showed a much higher SF concentration in the liver compared with an SF solution after the intravenous injection of SF-M6P-BSA nanoparticles into mice. A similarly high hepatic distribution of albumin nanoparticles was also reported by Kapoor *et al.* [23] and Santhi *et al.* [24].

Albumin nanoparticles could serve as a rational tumor-targeted drug delivery system because such nanoparticles would be expected to accumulate in solid tumors via the enhanced permeability and retention (EPR) mechanism when the size range is regulated at around 100 nm [25]. Recently, Qi *et al.* prepared glycyrrhetinic acid (GA)-modified HSA nanoparticles (GA-HSA nanoparticles) for the targeted delivery of doxorubicin to liver tumors [26]. The results of an *in vitro* cell uptake study and an *in vivo* biodistribution study revealed a promising new vehicle for targeting liver tumor chemotherapy in which doxorubicin-loaded GA-HSA nanoparticles increased cytotoxic activity in HepG2 cells *in vitro* and exhibited a much higher level of tumor accumulation in hepatoma (H22 cell)-bearing mice. Similarly, other groups prepared surface-modified HSA/BSA nanoparticles, such as hemato-porphyrin and particles modified with galactosamine, for the targeted delivery of anti-cancer drugs to hepatocellular carcinomas [27,28,29]. These suggest that albumin nanoparticles have the potential to function as a carrier for anti-cancer therapeutics, including liver tumors.

Watcharin *et al.* investigated the potential of HSA nanoparticles as contract agents for the detection of hepatocellular carcinoma by magnetic resonance imaging [30]. They prepared gadolinium diethylene–triaminepentaacetic acid-conjugated HSA nanoparticles and showed better optical visualization in an *in vitro* and *in vivo* study. This suggests that albumin nanoparticles have the potential for use as a promising diagnostic tool.

### 2.3. Fusion

The advancement of recombinant DNA technology enables two genetically fused protein molecules to be produced. In addition, rHSA has been successfully produced using *Pichia pastoris*, and the structural, physicochemical, and pharmacokinetic properties of rHSA were found to be identical to those of HSA derived from plasma [31,32], leading to an accelerated development of HSA fusion proteins. The main aim of genetic fusion with HSA is to improve the plasma retention of low molecular weight biological proteins, because HSA has a longer half-life (~19 days in humans [33,34]).

Our group designed and successfully prepared a fusion protein of thioredoxin-1 (Trx) and HSA (HSA-Trx), and the resulting HSA-Trx preserved the therapeutic efficacy of Trx (antioxidant and anti-inflammatory effect) [35]. Trx has great potential as a new therapeutic protein for the treatment of hepatic diseases [36,37,38], but its plasma half-life is too short to permit it to be used clinically [39]. On the other hand, HSA-Trx had a longer retention time in the blood circulation, compared with Trx [35]. In addition, the therapeutic efficacy of Trx was preserved in HSA-Trx, which showed useful effects as a therapeutic agent for treating acetaminophen-induced hepatitis [40]. These findings suggest that HSA-Trx is a promising therapeutic agent for the treatment of hepatic injuries.

Interferon (IFN)-α has stimulated a revolution in the treatment of chronic hepatitis C (HCV), and the current standard of HCV care is a combination of PEGylated IFN-α with ribavirin and boceprevir/telaprevir [41]. Albinterferon, a fusion of albumin and IFN-α2b, was developed as an alternative to PEGylated IFN, and has a long half-life (~8 days) and IFN-α-like phrmacodynamics properties [42]. Phase 3 studies of Albinterferon at a dose of 900 or 1200 μg injected at two-week intervals in combination with ribavirin showed an efficacy similar to PEGylated IFN-α for the treatment of chronic HCV genotype 1 or 2/3 [43,44]. However, increased rates of pulmonary adverse events were noted with Albinterferon, including interstitial lung disease, compared with those seen with PEGylated IFN-α. Many clinical trials are currently underway to evaluate the dosage regimen and new treatment options for this preparation, and findings are now being accumulated [45,46,47].

### 2.4. PEGylated Albumin

To further enhance the quality and efficiency of drug delivery substances, they are frequently modified with polyethylene glycol (PEG) [48]. There is now little doubt that PEGylation is useful and is in widespread use because it results in a prolonged half-life, a higher stability, and a lower immunogenicity. Thus, some PEGylated BSA or HSA preparations are created to take advantage of these characteristics [49,50,51], and the availability of such materials for drug delivery are currently being investigated [52,53,54].

NO delivery is expected to achieve therapeutic effects in hepatic injury, but NO gas as a therapeutic agent is limited because of its short half-life *in vivo*. Katsumi *et al.* used albumin as an NO-traffic material, and developed a PEGylated albumin-based NO donor in which 10 NO molecules were covalently bound to PEGylated BSA through *S*-nitrosothiol linkages (SNO-PEG-BSA), and SNO-PEG-BSA exceeded the extent of the release half-life of NO by compared to *S*-nitrocylated BSA (SNO-BSA) [55]. As a long-circulating NO donor, the administration of SNO-PEG-BSA to hepatic ischemia/reperfusion injury model mice clearly suppressed the hepatic injury [56]. Furthermore, they also developed PEGylated BSA with multiple reduced thiols (PEG-BSA-SH) [57]. PEG-BSA-SH was a highly effective scavenger of ROS *in vitro* and showed a long circulation time in the plasma after intravenous injection in mice. Furthermore, PEG-BSA-SH showed therapeutic potential for the treatment of fulminant hepatic failure that was induced by an intraperitoneal injection of d-galactosamine (d-GalN)/lipopolysaccharide (LPS) into mice [57]. These findings indicate that PEGylated albumin is a promising material for use in the treatment of hepatic injuries.

### 2.5. Albumin Dialysis

The molecular adsorbents recirculatory system (MARS^®^) is currently the most effective non-biological liver support device and can effectively remove protein-bound and water-soluble substances such as ammonia, bilirubin, bile acids, and medium- and short-chain fatty acids from the circulation [58]. This system is based on the ligand binding property of albumin, which removes albumin-bound toxins from the patient’s blood. Details of the principles of MARS^®^ and the results of clinical trials have been reported elsewhere [59,60,61]. To date, our research group identified key amino acid residues that contribute to the high affinity binding of bilirubin by a phage library and constructed an HSA mutant domain II with the objective of producing a therapeutic agent for use in the treatment of hyper-bilirubinemia in patients with impaired liver function [62,63]. This suggests that this HSA mutant domain II might be used in MARS^®^ applications where the removal of bilirubin and any other protein binding toxins from the body would be desirable.

## 3. Lactoferrin

**Figure 2:**
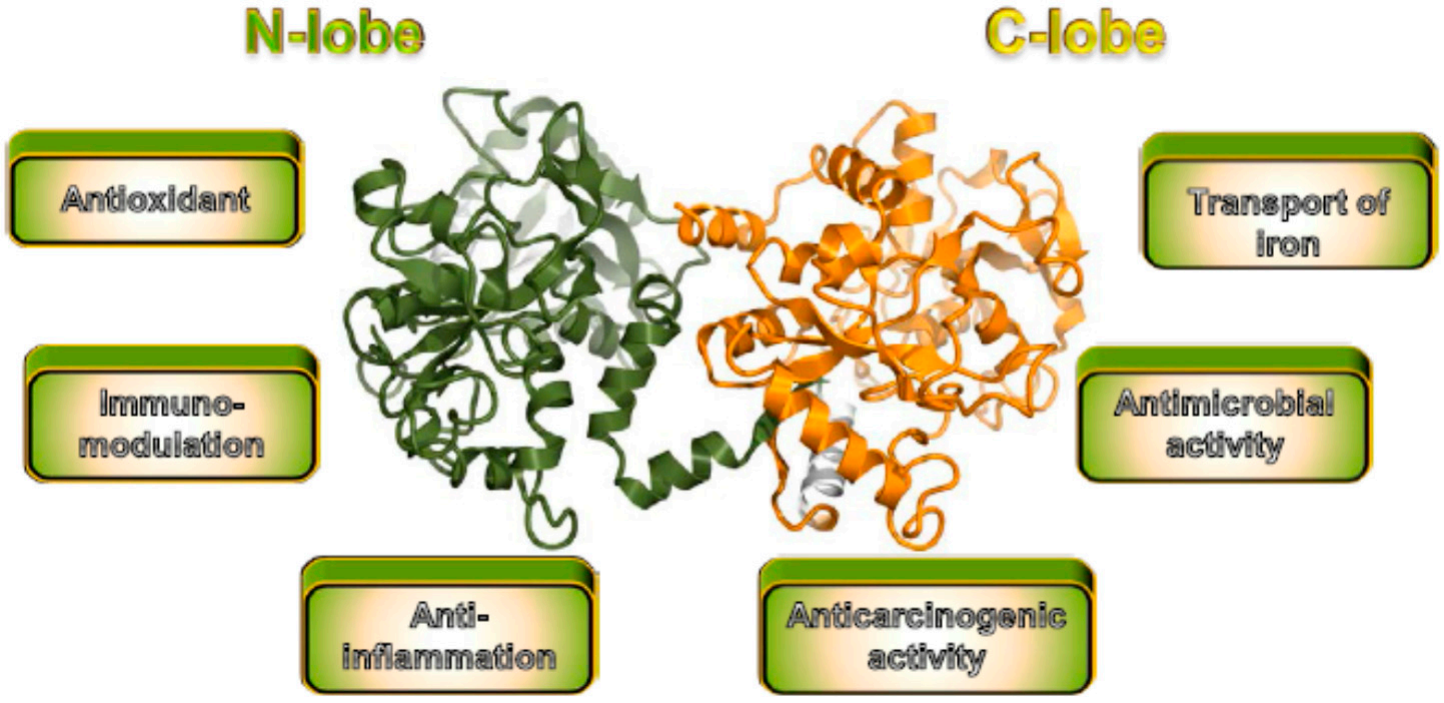
Crystal structure of bovine lactoferrin and its biological functions. Bovine lactoferrin consists of two lobes, an N-lobe (green) and a C-lobe (orange). The crystal structure was prepared using the CueMol software and the structural coordinates of PDB 1BLF.

Lactoferrin is a multiple bioactive protein that is found in mammalian milk and has protective effects ranging from anticancer to anti-inflammatory and antimicrobial (Figure 2) [64]. This multitude of biological activities suggests that lactoferrin is a potential drug candidate for the treatment of hepatitis.

### 3.1. Unmodified Lactoferrin

Yin *et al.* previously investigated the hepatoprotective effect of lactoferrin against acetaminophen-induced hepatitis [65]. The results showed that lactoferrin inhibited acetaminophen-induced liver sinusoidal endothelial cell damage and improved hepatic congestion [65]. Their group also reported a hepatoprotective effect of lactoferrin against concanavalin A-induced hepatitis, which mimics the pathophysiology of human viral and autoimmune hepatitis [66]. In addition, it was reported that the oral or intravenous administration of lactoferrin exhibited potent hepatoprotection against obstructive jaundiced rats [67], hepatic amoebiasis model hamster [68], d-GalN/LPS-induced hepatitis model mice [69], carbon tetrachloride-induced hepatitis model mice [69], and chemical-induce rat liver fibrosis [70]. Furthermore, lactoferrin also showed a protective effect on an HCV infection in hepatocytes in an *in vitro* study [71,72].

### 3.2. PEGylated Lactoferrin

Since the half-life of lactoferrin is too short to permit its use in protein-based products for use clinically, Sato and collaborators developed PEGylated lactoferrin in order to enhance plasma retention [73]. As expected, PEGylated lactoferrin had a comparable biological activity to unmodified lactoferrin and its plasma half-life was 8.7-fold longer than that of unmodified lactoferrin in rats [73]. Furthermore, this PEGylated lactoferrin was superior in hepatoprotection to unmodified lactoferrin through its anti-inflammatory properties in a rat model of acute liver injury induced by d-GalN/LPS or carbon tetrachloride [74,75]. These findings indicate that an increased plasma retention of lactoferrin has a massive potential in applications to therapy for hepatic injuries. Recently, their group developed lactoferrin-immunoglobulin G1 fragment crystallizable domain (Fc) fusion (Lf-Fc fusion), and its plasma half-life was found to be comparable to the PEGylated lactoferrin [76]. Thus, Lf-Fc fusion is expected in the future to have a beneficial effect on liver injury as a promising candidate drug.

### 3.3. Others

Lactoferrin has been used as a specific liver targeting ligand because it binds to asialoglycoprotein receptors with a high affinity [77]. Weeke-Klimp *et al.* prepared lactoferrin that was covalently coupled to stabilized plasmid lipid particles for non-viral gene delivery [78]. Its massive delivery in hepatocytes succeeded after systemic administration, but the researchers were not able to adequately transfect a reporter gene to hepatocytes *in vivo* [78]. Wei *et al.* developed lactoferrin-modified PEGylated liposomes for targeting hepatocellular carcinomas [79]. The results of *in vivo* imaging in HepG2 tumor-bearing mice showed that lactoferrin-modified PEGylated liposomes accumulated at higher levels in tumors than non-modified PEGylated liposomes [79]. These findings indicate that lactoferrin is a promising ligand for the design of delivery systems targeting the liver.

Kondapi *et al.* used lactoferrin as an anti-cancer drug carrier for cancer treatment due to the high expression of the lactoferrin receptor on the surface of metabolically active cancer cells [80,81,82]. The oral and intravenous administration of doxorubicin-loaded lactoferrin nanoparticles improved the efficacy and safety of doxorubicin for the treatment of hepatocellular carcinomas induced by diethylnitrosamine in rats compared to doxorubicin [81,82]. These findings indicate that lactoferrin is a promising anti-cancer drug carrier for use in hepatic cancer therapy.

### 3.4. Erythropoietin

**Figure 3:**
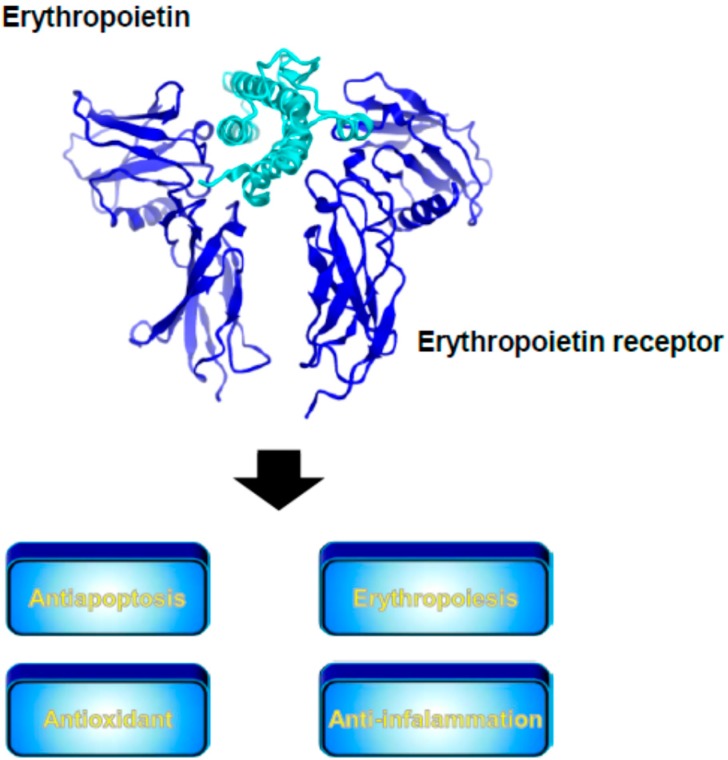
Crystal structure of erythropoietin (**aqua**) and erythropoietin receptor (**blue**) complex and its biological functions. The crystal structure was prepared using the CueMol software and the structural coordinates of PDB 1CN4.

Erythropoietin (EPO) is an acidic glycoprotein hormone with a molecular weight of approximately 30 kDa that is synthesized predominantly in the kidneys in response to erythropoietic stress such as tissue hypoxia [83]. Biologically, EPO has a central role in the formation of red blood cells (RBC) via binding to the EPO receptor (Figure 3). Thus, recombinant EPO was developed as an erythropoiesis-stimulating agent and used for the treatment of renal anemia. In addition to erythropoiesis, it is well known that EPO has multiple protective effects and exhibits antiapoptotic, antioxidant, and anti-inflammatory activities as well (Figure 3). Taking advantage of these biological activities of EPO, a number of researchers showed the protective effect of EPO in hepatic ischemia/reperfusion injury [84,85,86] that is mediated via the activation of the phosphatidylinositol-3 kinase/AKT/endotherial NO synthase pathway [84] or the inhibition of caspase-3 activation [85]. EPO also attenuates systemic ischemia/reperfusion injuries, such as liver and kidney, induced by resuscitation from a massive hemorrhage [87,88]. Furthermore, Ben-Ari *et al.* reported that EPO improved the survival and attenuated d-GalN/LPS-induced fulminant hepatic failure injury via upregulation of the EPO receptor and phosphatidylinositol-3 kinase [89,90]. These results suggest the potential importance of using EPO in the treatment of acute hepatic injuries.

In animal studies, it was clearly shown that systemically administrating EPO increased liver regeneration and enhanced survival after a partial hepatectomy [91,92,93]. In addition, granulocyte colony stimulating factor (G-CSF) has been shown to increase survival in patients with acute or chronic liver failure [94,95]. Therefore, it would be expected that the exogenous co-administration of EPO and G-CSF would improve liver regeneration and survival after a subtotal hepatectomy. In fact, Vassiliou *et al.* reported that perioperatively administered EPO and G-CSF enhanced liver regeneration in a rat study [96]. More recently, Kedarisetty *et al.* performed a prospective study, a single-center randomized trial, involving a series of patients with decompensated cirrhosis who were randomly assigned to groups and given G-CSF and EPO (darbopoietin α) or a placebo [97]. The results showed that a larger proportion of patients who received a combination of G-CSF and EPO survived compared to patients given only placebos. These animal and human studies indicate that the administration of EPO or the co-administration of EPO and G-CSF is a promising therapeutic strategy for patients with decompensated cirrhosis who cannot survive without a liver transplant.

### 3.5. α_1_-Acid Glycoprotein

**Figure 4:**
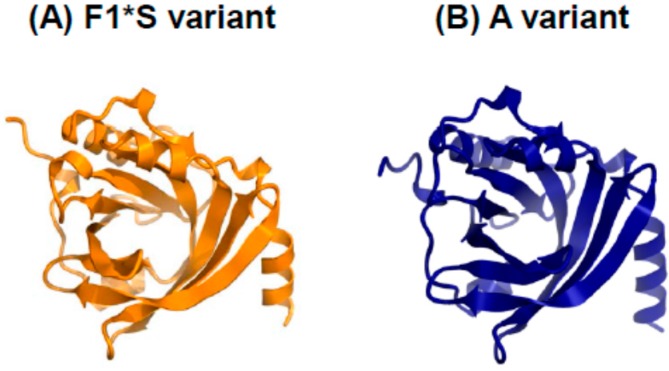
Crystal structures of the human AGP F1*S (**A**) and A variants (**B**) at a resolution of 1.8 and 2.1 Å, respectively. Both illustrations were produced with CueMol using the atomic coordinates from the Protein Data Bank, 3KQ0 for (**A**) and 3APX for (**B**).

α_1_-Acid glycoprotein (AGP) is an acute phase protein in the blood. AGP is comprised of 183 amino acid residues and contains five N-linked oligosaccharides, with a molecular weight of approximately 44 kDa [98]. AGP exists as two main genetic variants, namely F1*S and A variants (Figure 4), and the molar ratio of the F1*S and A variant in the blood typically ranges from 3:1 to 2:1. Although the detailed biological functions of AGP have not been elucidated completely, one of its physiological roles appears to involve immunomodulating effects [98]. Kagaya *et al.* reported that AGP inhibited the cell death of rat primary hepatocytes that had been treated with a chemical toxin (bromobenzene) [99]. In addition, Van Molle *et al.* found that AGP inhibits apoptosis of hepatocytes induced by TNF/galactosamine and TNF/actinomycin D in mice via suppressing the activation of caspase 3 and 7, which is a key factor in inducing apoptosis [100,101]. Furthermore, Kuzuhara *et al.* demonstrated that treatment with AGP reduced multifocal necrosis in the liver in concanavalin A-induced hepatitis model mice [102]. These findings indicate that AGP potentially possesses anti-apoptosis or cytoprotective effects for hepatocytes and can be used as a potential new therapeutic intervention in the treatment of hepatic injury.

### 3.6. Gelatin

Gelatin is a denatured protein that is made of animal collagen; it is clinically used as a plasma expander and included as a stabilizer in a number of protein formulations due to its biocompatibility, biodegradability, and low cost [103]. These advantages of gelatin led to its application as a material for drug delivery. Gelatin-based nanoparticles are a promising carrier system for delivering hydrophilic and hydrophobic drugs, proteins, and vaccine [103]. Hoffmann *et al.* prepared NF-κB inhibiting decoy oligodeoxynucleotide-loaded gelatin nanoparticles, and clearly showed their therapeutic effects against d-GalN/LPS-induced fulminant hepatic failure injury and concanavalin A-induced hepatitis [104]. Moreover, some researchers developed surface-modified, gelatin-based nanoparticles with site-specific ligands, such as a carbohydrate and a peptide [103], and achieved the site-specific delivery of drugs to macrophages in the liver [105,106] and hepatocarcinomas [107].

### 3.7. Hemoglobin

**Figure 5:**
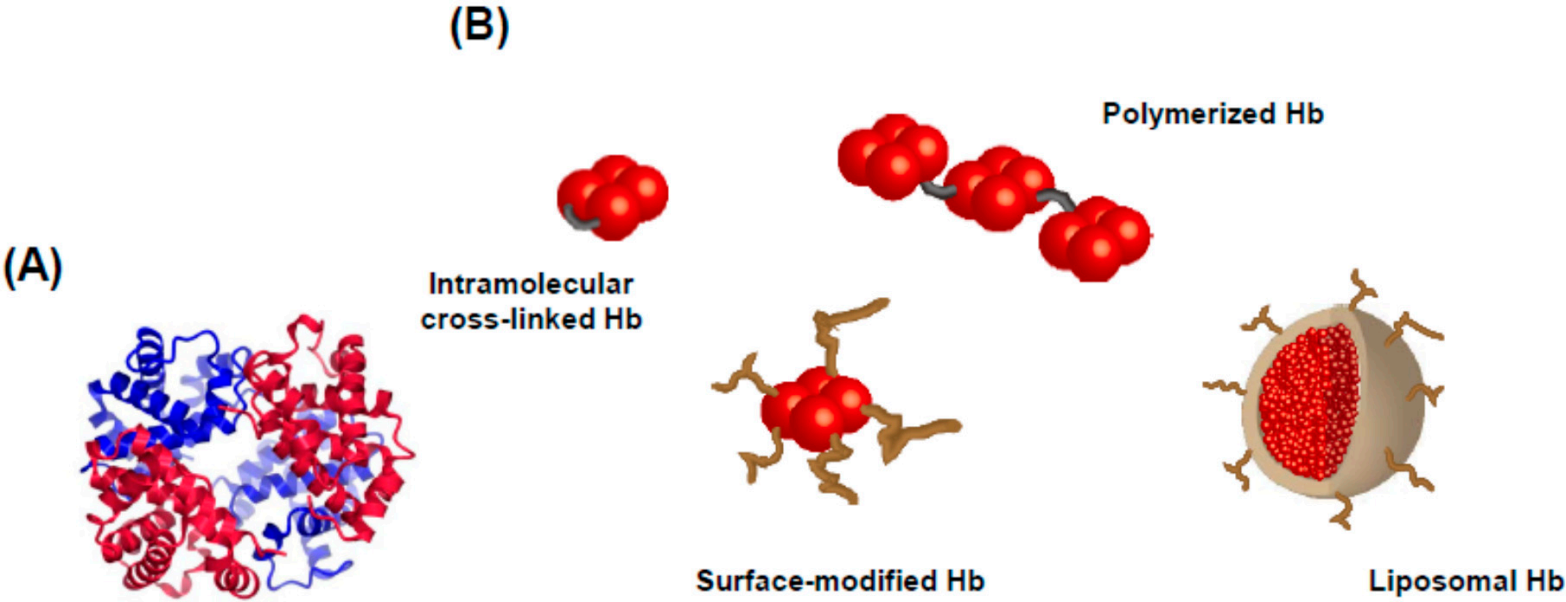
(**A**) Crystal structure of deoxy hemoglobin at a resolution of 1.74 Å. (**B**) Schematic representation of acellular type and cellular type of hemoglobin-based oxygen carriers. Hemoglobin consists of four subunits, two alpha subunits (**blue**) and two beta subunits (**red**). The crystal structure was prepared using the CueMol software and the structural coordinates of PDB 2HHB.

Hemoglobin (Hb) consists of four subunits (two alpha subunits and two beta subunits) and four heme moieties (Figure 5A), and in red blood cells (RBC) is responsible for delivering oxygen from respiratory organs to anaerobic (periphery) tissues. Thus, RBC transfusions are clinically used for resuscitation from hemorrhagic shock as the gold standard therapy. However, since donated RBCs for blood transfusions can only be stored for a short period (three weeks in Japan), the acellular type or cellular type of Hb-based oxygen carriers (surface-modified Hb [108], intramolecularly cross-linked Hb [109] and polymerized Hb [110], and liposomal Hb [111]: Figure 5B) have been developed as RBC substitutes in order to produce superior characteristics such as the absence of viral contamination and a long-term storage period for donated RBC. Although these formulations were developed as oxygen carriers, some researchers focused on its effective carbon monoxide (CO) delivery and demonstrated their potential as a CO carrier [112,113]. Sakai *et al.* administered CO-bound liposomal Hb to hemorrhagic shock model rats as a resuscitation fluid [114]. The results of this study showed that CO-bound liposomal Hb transfusion functioned as a resuscitation fluid as well as RBC transfusion and attenuated the hepatic injury induced by systemic ischemia/reperfusion injury [114]. Similar effects were observed when RBCs were used as a CO carrier [115,116].

Since native Hb generated from lysed RBCs is scavenged by macrophages via the CD163 scavenging receptor [117,118], some researchers have used Hb as a macrophage-specific ligand. Zhang and Palmer developed a liposome surface conjugate with Hb and demonstrated the feasibility of using this carrier in macrophage-targeted drug delivery [119]. Adamson and collaborators synthesized a Hb-ribavirin conjugate for use in the treatment of viral hepatitis [120,121]. They clearly showed the selective uptake by CD163-expressed cells *in vitro* and targeted ribavirin delivery to the liver in an *in vivo* study [120,121]. Furthermore, the Hb–ribavirin conjugate inhibited viral replication *in vitro* and altered the course of hepatitis virus infections *in vivo* as demonstrated by prolonged survival, improved behavior, and reduced signs of histologically evident disease in mice [121]. CD163 is now recognized as one of the useful targets for the therapy of hepatitis because it indirectly contributes to the anti-inflammatory response [122]. These results indicate that Hb has the potential to function as both a novel carrier of CO and as CD163 in a macrophage-specific ligand.

## 4. Conclusions

Numerous protein-based products have been developed in attempts to achieve their clinical use as pharmaceutical products, but fewer protein-based products have been approved for clinical use than other low-molecule drugs. Recent advances in scientific technologies such as recombinant protein engineering, biochemical analysis, and analytical instrumentation technique provide novel functional aspects and insights into not only proteins but also the liver to further develop innovative protein-based products for the treatment of hepatic diseases. In the near future, it is expected that novel protein-based products, similar to albumin–paclitaxel nanoparticles (Abraxane^®^), will be clinically developed for use in treating a variety of hepatic diseases.
